# Discrepancy between *Mtb*-specific IFN-γ and IgG responses in HIV-positive people with low CD4 counts

**DOI:** 10.1016/j.ebiom.2023.104504

**Published:** 2023-03-02

**Authors:** Maphe Mthembu, Kathryn A. Bowman, Leela R.L. Davies, Sharon Khuzwayo, Lusanda Mazibuko, Thierry Bassett, Dirhona Ramjit, Zoey Mhlane, Farina Karim, Galit Alter, Thumbi Ndung'u, Emily B. Wong

**Affiliations:** a Africa Health Research Institute, Durban, South Africa; b University of KwaZulu-Natal, Durban, South Africa; c Ragon Institute of MGH, MIT and Harvard, Cambridge, MA, USA; d HIV Pathogenesis Programme, The Doris Duke Medical Research Institute, University of KwaZulu-Natal, Durban, South Africa; e Division of Infection and Immunity, University College London, London, United Kingdom; f Division of Infectious Diseases, Heersink School of Medicine, University of Alabama at Birmingham, AL, USA

**Keywords:** QuantiFERON assay, Latent TB infection, HIV, CD4 count quartiles

## Abstract

**Background:**

Tuberculosis (TB) is a leading infectious cause of death worldwide and treating latent TB infection (LTBI) with TB preventative therapy is a global priority. This study aimed to measure interferon gamma (IFN-γ) release assay (IGRA) positivity (the current reference standard for LTBI diagnosis) and *Mtb-*specific IgG antibodies in otherwise healthy adults without HIV and those living with HIV (PLWH).

**Methods:**

One-hundred and eighteen adults (65 without HIV and 53 antiretroviral-naïve PLWH), from a peri-urban setting in KwaZulu-Natal, South Africa were enrolled. IFN-γ released following stimulation with ESAT-6/CFP-10 peptides and plasma IgG antibodies specific for multiple *Mtb* antigens were measured using the QuantiFERON-TB Gold Plus (QFT) and customized Luminex assays, respectively. The relationships between QFT status, relative concentrations of anti-*Mtb* IgG, HIV-status, sex, age and CD4 count were analysed.

**Findings:**

Older age, male sex and higher CD4 count were independently associated with QFT positivity (*p* = 0.045, 0.05 and 0.002 respectively). There was no difference in QFT status between people with and without HIV infection (58% and 65% respectively, *p* = 0.06), but within CD4 count quartiles, people with HIV had higher QFT positivity than people without HIV (*p* = 0.008 (2nd quartile), <0.0001 (3rd quartile)). Concentrations of *Mtb*-specific IFN-γ were lowest, and relative concentrations of *Mtb*-specific IgGs were highest in PLWH in the lowest CD4 quartile.

**Interpretation:**

These results suggest that the QFT assay underestimates LTBI among immunosuppressed people with HIV and *Mtb*-specific IgG may be a useful alternative biomarker for *Mtb* infection. Further evaluation of how *Mtb*-specific antibodies can be leveraged to improve LTBI diagnosis is warranted, particularly in HIV-endemic areas.

**Fundings:**

NIH, AHRI, SHIP: SA-MRC and SANTHE.

**Research in context**

**Evidence before this study**

*Mtb*-specific IFN-γ production as measured by IGRA is the current gold standard for determining latent TB infection. However, since these tests measure immunoreactivity to *Mtb* peptides, they are indirect measures of *Mtb* infection and their performance characteristics are impacted by co-infections and comorbidities that influence immune responses, including HIV. Recently, a human phenotype has been defined in people who are highly exposed to *Mtb* but consistently test negative for evidence of *Mtb* infection by IGRA and tuberculin skin test (TST). These individuals have been observed to have a unique profile of *Mtb*-specific antibodies when compared to the classical IGRA positive LTBI group, suggesting that *Mtb*-specific antibodies may identify additional people with a history of *Mtb* infection or exposure when compared to IGRA alone. Comparison of IGRA and *Mtb*-specific antibodies in people living with HIV (PLWH) has not previously been performed.

**Added value of this study**

Here, we concurrently assessed *Mtb*-specific IFN-γ production and IgG in a cohort of 118 well-defined individuals living with and without HIV from KwaZulu-Natal, South Africa, a highly TB endemic area. We found a discrepancy between *Mtb*-specific IFN-γ and *Mtb*-specific IgG, particularly in PLWH showing low CD4 cell counts. Notably people with the lowest CD4 counts had the highest relative concentrations of *Mtb*-specific IgG in the plasma, and the lowest rates of QTF positivity.

**Implications of all the available evidence**

IGRAs may underestimate *Mtb* infection status, especially in people with HIV infection or who have T cell depletion or dysfunction. *Mtb*-specific IgG antibodies indicate development of a B cell response to *Mtb* and upon further investigation may have promise as an alternative biomarker of TB immunoreactivity that does not depend on T cell function.

## Introduction

Tuberculosis (TB) is the leading cause of death for PLWH. Despite recent improvements in TB diagnostics and increasing access to anti-tubercular therapy, TB mortality remains high with an estimated annual death rate of 15% among the 10 million people who annually are diagnosed with TB, and with 21% of those deaths attributable to co-infection with HIV.^1^ An important challenge to eliminating TB is that *Mycobacterium tuberculosis* (*Mtb*) infection presents as a dynamic continuum, ranging from rapidly cleared *Mtb* infection to active TB (ATB) disease. Currently available diagnostic tools are imperfect and are incapable of accurately identifying the different stages of TB pathogenesis.^2^^,^^3^ Considering that there are no direct tests of mycobacterial burden, people who have immunological evidence of *Mtb* infection and who are clinically free of disease have historically been considered to have “latent TB infection” (LTBI), although use of this term is increasingly criticized because of its inherent imprecision.^4^, ^5^, ^6^ It has been estimated that 30% of people in the world have LTBI and PLWH who have LTBI are at higher risk of progressing to active disease.^1^^,^^7^ Preventative therapies, including 6–9 months of isoniazid and more recently tested shorter courses of multiple drugs, like 3 months of rifapentine and isoniazid (3HP), have been successful in reducing the short-term risk of progression to ATB in PLWH.^8^, ^9^, ^10^

The impact of TB prevention therapies has been limited by the difficulty of diagnostic testing for LTBI. The tuberculin skin test (TST) uses purified protein derivative (PPD) from *Mtb* and has significant rates of cross-reactivity with the *M. bovis* BCG strain used to vaccinate infants in TB endemic countries.^5^ *Mtb*-specific IGRA tests have improved specificity for *Mtb* and are therefore the current gold standard for determining *Mtb* infection status, however, both tests (TST and IGRA) rely on T cell immune responses^4^^,^^5^^,^^11^ and therefore fail to determine whether viable *Mtb* infection is present. Additionally, both TST and IGRA perform less well in immunocompromised people with decreased T cell function, including PLWH.^12^^,^^13^ Moreover, the indirect nature of these tests limits the field's ability to precisely define the pathogenesis of LTBI,^14^ which is required to design more effective TB prevention strategies that are targeted to the optimal stage in the spectrum of TB disease.

In this study, we sought to measure rates of, and characteristics associated with LTBI in a peri-urban setting in KwaZulu-Natal, South Africa by comparing *Mtb*-specific IFN-γ and *Mtb*-specific IgG antibodies in otherwise healthy adults living with and without HIV.

## Methods

### Study population and clinical characteristics

Data and plasma from participants who were screened for the Phefumula cohort, a research bronchoscopy study described previously in Muema et al., 2020^15^ and Khuzwayo et al., 2021,^16^ were used for this retrospective analysis. Adult males or females (self-reported gender) living with and without HIV, between 18 and 50 years, who were otherwise healthy (no chronic medical conditions, no current or past tobacco use, no current TB symptoms) were recruited from KwaDabeka Community Health Clinic in peri-urban KwaZulu-Natal, South Africa. The HIV status of all participants was determined by fourth generation enzyme linked-immunosorbent assay (ELISA) testing and HIV RNA plasma viral load (Sigma–Aldrich). Participants living with HIV were newly diagnosed and had not initiated antiretroviral therapy (ART) at the time of enrolment; on the day of enrolment they received immediate referral for ART-initiation according to South African guidelines. No longitudinal data was collected after ART initiation. BCG and influenza vaccination status were not recorded.

### Ethics

All participants provided written informed consent for participation. Study protocols were approved by the University of KwaZulu-Natal Biomedical Research Ethics Committee (BREC, protocol BF503/15), the Partners Institutional Review Board and the University of Alabama Institutional Review Board.

### Measurement of *Mtb*-specific IFN-γ

The QuantiFERON-TB Gold Plus (QFT-Plus) assay (Qiagen) was conducted as per manufacturer's instructions. Detailed information on the list of reagents used for this study are summarised in Supplementary Table 1. Briefly, whole blood was collected into QFT tubes at room temperature, thoroughly mixed and then incubated at 37°C for 20 hours to allow stimulation by TB antigens (early secretory antigenic target 6 [ESAT6] and culture filtrate protein 10 [CFP10]). After centrifugation at 2500 RPM for 15 minutes, supernatant was collected and 50 μL used for IFN-γ ELISA, performed according to the manufacturer's instructions. The results were analysed using the QFT-Plus analysis software v2.71.2 (Qiagen). A positive QuantiFERON was defined by IFN-γ produced in response to TB1 or TB2 minus the NIL tube (negative control, ≤0.8 IU/mL) ≥ 0.35 IU/mL.

### Measurement of *Mtb*-specific IgG

#### Antigen selection and source

PPD was obtained from Staten Serum Institut. Antigen 85 complex (Ag85), ESAT-6/CFP-10, alanine- and proline-rich secreted protein (Apa), groES, alpha-crystallin (also known as heat shock protein X (HspX)), and lipoarabinomannan (LAM) were obtained from BEI Resources (NR-14855, NR-49424, NR-49425, NR-49428, NR-14862, NR-14861, NR-14860, NR-14848, respectively). The 6 well-characterized immunogenic *Mtb* antigens were selected as those that are commercially available and include both surface and intracellular bacterial components that are known *Mtb* antibody targets. Influenza hemagglutinin from A/New Caledonia/20/1999 and B/Brisbane/60/2008 (ImmuneTech) was included as an internal control.

#### Customized multiplex luminex assay

*Mtb*-specific IgG was measured using a Luminex isotype assay described by Lu et al., 2019.^17^ Briefly, we bound Luminex MagPlex® carboxylated beads (Luminex) with *Mtb*-specific antigens and non-*Mtb* related antigen using N-Hydroxysuccinimide (NHS)-ester linkages following manufacturer's recommendations. Protein antigens were coupled to Magplex carboxylated beads using 1-ethyl-3-(3-dimethylaminopropyl) carbodiimide hydrochloride and N-hydroxysulfosuccinimide) according to the manufacturer's recommendations. LAM was modified by 4-(4,6-dimethoxy[1,3,5]triazin-2-yl)-4-methyl-morpholinium prior to conjugation. Multiplexed antigen-coupled beads were incubated with sample (supernatant from the QFT-Plus NIL tube) at a 1:100 dilution in assay buffer (PBS with 0.1% BSA and 0.05% Tween) for 18 hours. IgG was detected using phycoerythrin-conjugated mouse antibodies against human antibody subclasses (Southern Biotech 9040-09). Secondary incubations were performed over 2 hours. Analysis was performed on an iQue Screener PLUS using ForeCyt software (Intellicyt). The relative concentration of IgG was expressed as Median Fluorescence Intensity (MFI) and absolute concentrations of these were not calculated. All assays were performed in duplicates.

### Quantitative measurement of total IgG

ELISA plates were coated overnight with anti-human IgG (Sigma Aldrich I5260) at 1:5000 in PBS, then washed 3 times in PBS-0.05% Tween (PBST) and blocked with 5% BSA in PBS. Primary incubation was performed with serum diluted at 1:1 000 000 for 2 hours at room temperature, then plates were washed 3 times in PBST. Secondary incubation was performed with 1:20 000 dilution of HRP-conjugated anti-human IgG antibody (Sigma Aldrich SAB701283) for 1 hour at room temperature, then plates washed 3 times in PBST. Plates were developed with 1-Step™ Ultra TBM-ELISA substrate solution (Thermo Fisher) and stopped with equal volume 1N H_2_SO_4_. Absorbance was read at 450 nm with reference 570 nm. GammaGard liquid IVIG was used as a quantitative standard.

### Reagent validation

All reagents used in this study were obtained commercially and were not independently validated in our laboratory.

### Statistics

Statistical analyses and graphical representations were performed using GraphPad Prism v9.2.0 (GraphPad Software) and Stata/SE v17.0 (StataCorp LLC). Non-parametric tests (Fishers exact and Chi-squared tests for single or multiple categorical comparisons and Mann–Whitney *U* and Kruskal–Wallis tests for single and multiple comparisons of continuous dependent variables) were used to avoid assumptions about data normality. Logistic regression was employed for multivariate analysis of QFT status as a binary dependent variable. For single comparisons a significance threshold of *p* < 0.05 was used. For measures of correlations between study variables, Benjamini-Hochberg correction tests were applied to account for multiple comparisons.

### Role of funders

Funders had no practical involvement in the study design, samples collection, data analysis, data interpretation or in the writing of the publication.

## Results

### Characteristics of study participants

One hundred and eighteen otherwise healthy adults between the ages of 18–50 years were enrolled. Sixty-five of them people living without HIV and 53 were PLWH (Table 1). To meet enrolment criteria, participants had to be feeling well, have no prior history of TB or other co-morbidity. All the PLWH were newly diagnosed and antiretroviral therapy (ART)-naïve. The majority of participants were female (69%), with sex distribution balanced between the PLWH (71% females) and people without HIV (66% females) (*p* = 0.6969). The PLWH was slightly older than the people without HIV (*p* = 0.0234). CD4 T-cell count was measured for all study participants and the median (interquartile range, IQR) for the PLWH and people without HIV was 358 cells/mm^3^ (IQR: 210–575) and 870 cells/mm^3^ (IQR: 710–1201), respectively (*p* < 0.0001). Viral load for the PLWH group was high (median of 123 919 copies/mL (IQR: 10 600–2 124 982)), as expected for an ART-naïve population. Age and CD4 count values of all participants were stratified into quartiles and the number of participants in each quartile for the PLWH and those without HIV is shown in Table 1.

**Table 1:** Demographic and clinical characteristics.

Characteristics	All participants	HIV-Negative	HIV-Positive	*p* value∗
N	118	65	53	–
Sex				0.6969
Females n (%)	81 (69%)	43 (66%)	38 (72%)	
Males n (%)	37 (31%)	22 (34%)	15 (28)	
Age median (IQR)	30 (24–35)	27 (23–33)	31 (27–35)	0.0234
1st quartile (18–24) n	30	21	10	–
2nd quartile (25–30) n	34	19	15	–
3rd quartile (31–34) n	31	14	17	–
4th quartile (35–49) n	23	11	12	–
CD4 T cell count (cells/mm^3^) median (IQR)	685 (431–947)	870 (710–1201)	358 (210–575)	<0.0001
1st quartile (0–428) n (median)	30 (212)	0 (−)	30 (212)	–
2nd quartile (429–685) n (median)	29 (582)	14 (640.5)	15 (529)	0.0103
3rd quartile (686–923) n (median)	29 (797)	21 (795)	8 (801)	0.8667
4th quartile (923–2043) n (median)	30 (1233)	30 (1233)	0 (−)	–
Viral load (copies/mL) median (IQR)	–	0	123,919 (10,600–2124 982)	–

Data shown in median and interquartile range (IQR) unless stated otherwise.

Mann–Whitney *U* test was used to calculate significance between the two study groups. In the comparison for gender distribution between the groups, Chi-square test was used.

*p* value∗ for statistical significance between HIV-negative and HIV-positive people.

The “-” means the test was not done.

IQR, interquartile range.

### ESAT-6/CFP-10-specific IFN-γ is associated with higher CD4 cell count

**Fig. 1:**
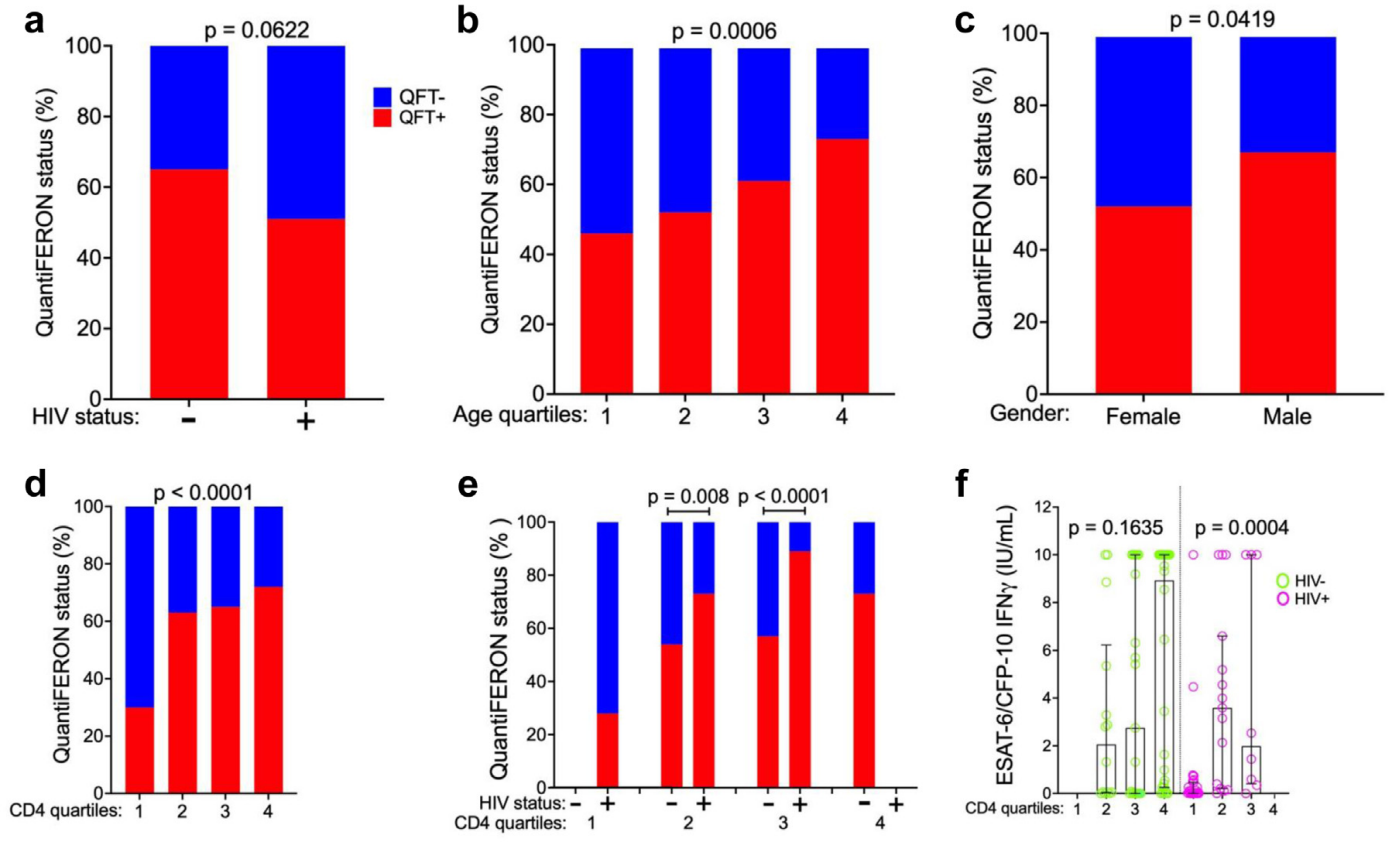
**Factors associated with QuantiFERON positivity.** QuantiFERON test results expressed as percentage (%) positive (red bars) or negative (blue bars) in 118 participants, stratified according to **a.** HIV status (people without HIV (n = 65), people with HIV (n = 53), Fischer's exact test of difference between groups), **b.** age quartiles (Chi squared test of difference across quartiles), **c.** sex (Fisher's exact test), **d.** CD4 count quartiles (Chi-squared test) and **e.** by HIV status within CD4 quartiles (Fishers Exact test was used to test for difference between HIV groups within comparable CD4 count quartiles). **f.** ESAT-6/CFP-10 specific IFN-γ concentration (IU/mL) among people without HIV (green) and people with HIV (pink) by CD4 counts quartile (Fischer's exact test).

We first aimed to determine the rates of QFT positivity within our study population. All study participants were screened for latent TB infection using the QFT-Gold plus test. Fifty-eight percent of participants had a positive QFT test with a trend towards higher positivity among the people without HIV (65%) when compared to PLWH (51%) (*p* = 0.06) (Fig. 1a). Stratifying participants by age quartiles showed a significant increase in QFT positivity with increasing age (*p* = 0.006) (Fig. 1b). Males had higher QFT positivity compared to females (*p* = 0.0419) (Fig. 1c). Stratification by CD4 cell count quartiles showed that QFT positivity increased significantly with CD4 cell count (*p* < 0.0001) (Fig. 1d).

We hypothesized that people with HIV would have equivalent or higher rates of latent TB infection compared to people without HIV and that CD4 T-cell deficiency led to the lower rates of positive QFT testing observed in people with HIV. To test this hypothesis, we stratified the entire cohort into quartiles by CD4 T-cell count. Quartile 1 included only people with HIV and quartile 4 included only people without HIV; Quartiles 2 and 3 each contained both people with and without HIV. When compared within CD4 quartile, people with HIV had higher QFT positivity than people without HIV (*p* = 0.008, and *p* < 0.0001 for quartiles 2 and 3 respectively, Fig. 1e). Among participants without HIV, there were no differences in the production of IFN-γ across CD4 count quartiles (*p* = 0.1635, Fig. 1f). However, in people with HIV there was significantly lower production of IFN-γ in the lowest CD4 count quartile (*p* = 0.0004, Fig. 1f and Supplementary Fig. 1a). In a multivariate analysis that accounted for age, sex, HIV status and CD4 count, older age (*p* = 0.045), male sex (*p* = 0.05) and higher CD4 cell count (*p* = 0.002) were independently associated with QFT positivity and HIV-status was not (Table 2). Taken together, these data indicate that CD4 count is associated with lower quantitative IFN-γ release in individuals living with HIV and may underlie the lower rates of QFT positivity observed in this population.

**Table 2:** Unadjusted and adjusted multivariate logistic regression model for QuantiFERON positivity.

Variables	Unadjusted Model		Adjusted Model	
	Odds Ratio (CI)	*p* value	Odds Ratio (CI)	*p* value
Age	1.05 (1.00–1.11)	0.051	1.06 (1.00–1.13)	0.045
Sex				
Female	1 (Reference)		1 (Reference)	
Male	1.86 (0.83–4.20)	0.133	2.36 (1.00–5.60)	0.050
HIV Status				
Negative	1 (Reference)		1 (Reference)	
Positive	0.57 (0.27–1.19)	0.135	1.38 (0.44–4.36)	0.581
CD4 Count	1.00 (1.01–1.02)	0.010	1.01 (1.00–1.00)	0.015
CD4 Count tertile:				
Low	1 (Reference)		1 (Reference)	
Medium	2.56 (1.03–6.37)	0.043	6.25 (1.44–27.16)	0.014
High	4.64 (1.77–12.19)	0.002	15.43 (2.73–87.13)	0.002

Hosmer–Lemeshow goodness of fit: 0.9659.

Unadjusted model *p* values are calculated from univariate analysis and adjusted *p* values are calculated from multivariate logistic regression model.

CI, Confidence interval.

### ESAT-6/CFP-10-specific antibody responses are higher in people with HIV and low CD4 cell counts

**Fig. 2:**
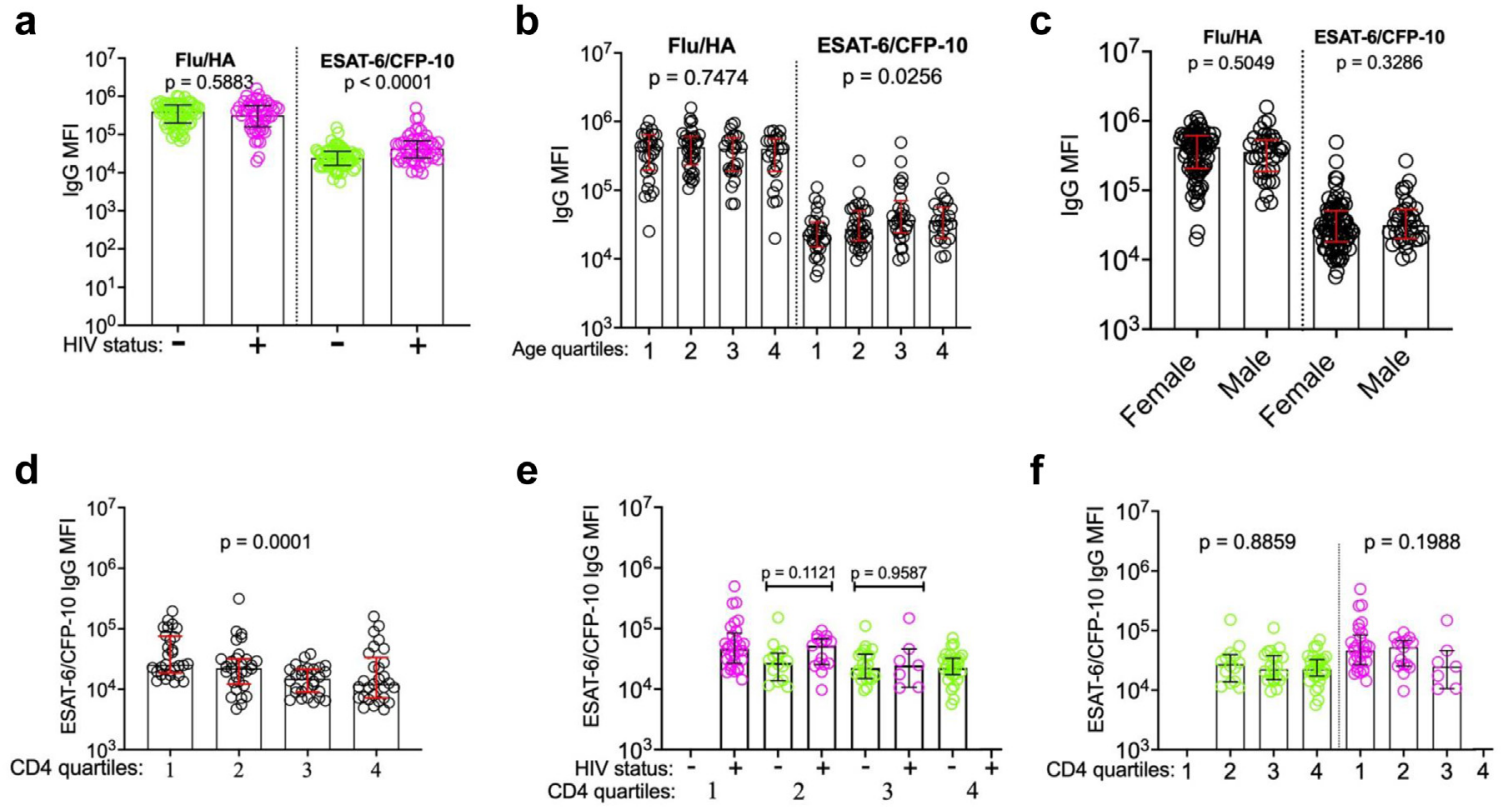
**Factors associated with ESAT-6/CFP-10-specific total IgG relative concentration**. **a.** Relative concentrations (MFI) of influenza hemagglutinin (HA)- and ESAT-6/CFP-10-specific IgG in 115 participants, stratified according to **a.** HIV status (people without HIV (n = 64, green), people with HIV (n = 51, pink), Mann–Whitney *U* test of difference between groups), **b.** age quartiles (Kruskal–Wallis test for difference across quartiles), **c.** sex (Mann–Whitney *U* test). **d.** Relative concentration of ESAT-6/CFP-10 total IgG (MFI) stratified by CD4 count quartiles (Kruskal–Wallis test), **e.** HIV status within CD4 quartiles (Mann–Whitney *U* test), **f.** among people without and with HIV, by CD4 quartile (Fischer's exact test).

Since IGRAs rely on functional CD4 T-cell responses, we next asked whether antibody responses could provide a more sensitive marker of *Mtb* exposure or infection in PLWH. We measured the *Mtb*-specific IgG responses in the same individuals using a customized Luminex assay to characterize the *Mtb*-specific antibody response.^17^ We focused on antibodies to ESAT-6/CFP-10 as these are relatively specific to *Mtb* compared with environmental mycobacteria.^18^ ESAT-6/CFP-10-specific IgG was analysed according to HIV status, age, sex and CD4 count. ESAT-6/CFP-10-specific IgG relative concentrations were significantly higher in people with HIV (*p* < 0.0001) compared to people without HIV (Fig. 2a). In contrast, relative concentrations of influenza hemagglutinin (HA)-specific IgG (non-*Mtb* control) did not differ by HIV status (*p* = 0.5883) (Fig. 2b). Relative concentrations of ESAT-6/CFP-10-specific IgG, but not of Flu/HA IgG, significantly increased with age quartiles (*p* = 0.0256, *p* = 0.7474 respectively) (Fig. 2b) although relative concentration of ESAT-6/CFP-10-IgG did not correlate with age (Supplementary Fig. 1b). Relative concentrations of ESAT-6/CFP-10 IgG and Flu/HA IgG did not differ between males and females (*p* = 0.3286, *p* = 0.5049, respectively) (Fig. 2c).

We next asked whether *Mtb*-specific IgG responses differed across CD4 T-cell count quartiles. ESAT-6/CFP-10-specific IgG relative concentrations differed significantly by CD4 count quartiles (*p* = 0.0001), with participants in the lowest CD4 count quartile having the highest IgG relative concentrations (Fig. 2d and Supplementary Fig. 1c). In contrast to the pattern observed for ESAT-6/CFP-10 IFN-γ, HIV status did not significantly affect the expression of ESAT-6/CFP-10-specific IgG when people without and people with HIV in the 2nd and 3rd CD4 cell count quartiles were compared (*p* = 0.1121 and *p* = 0.9587 respectively) (Fig. 2e). Within both the sub-populations of people with and without HIV, there was no significant difference in ESAT-6/CFP-10-specific IgG between CD4 count quartiles (*p* = 0.8859 and *p* = 0.1988, Fig. 2f). Thus, while ESAT6/CFP10-specific IgG is higher in individuals with HIV in our cohort, this association is unrelated to CD4 T-cell count.

### Additional anti-*Mtb* specific IgG antibody responses are also highest in people with HIV and low CD4 cell counts

**Fig. 3:**
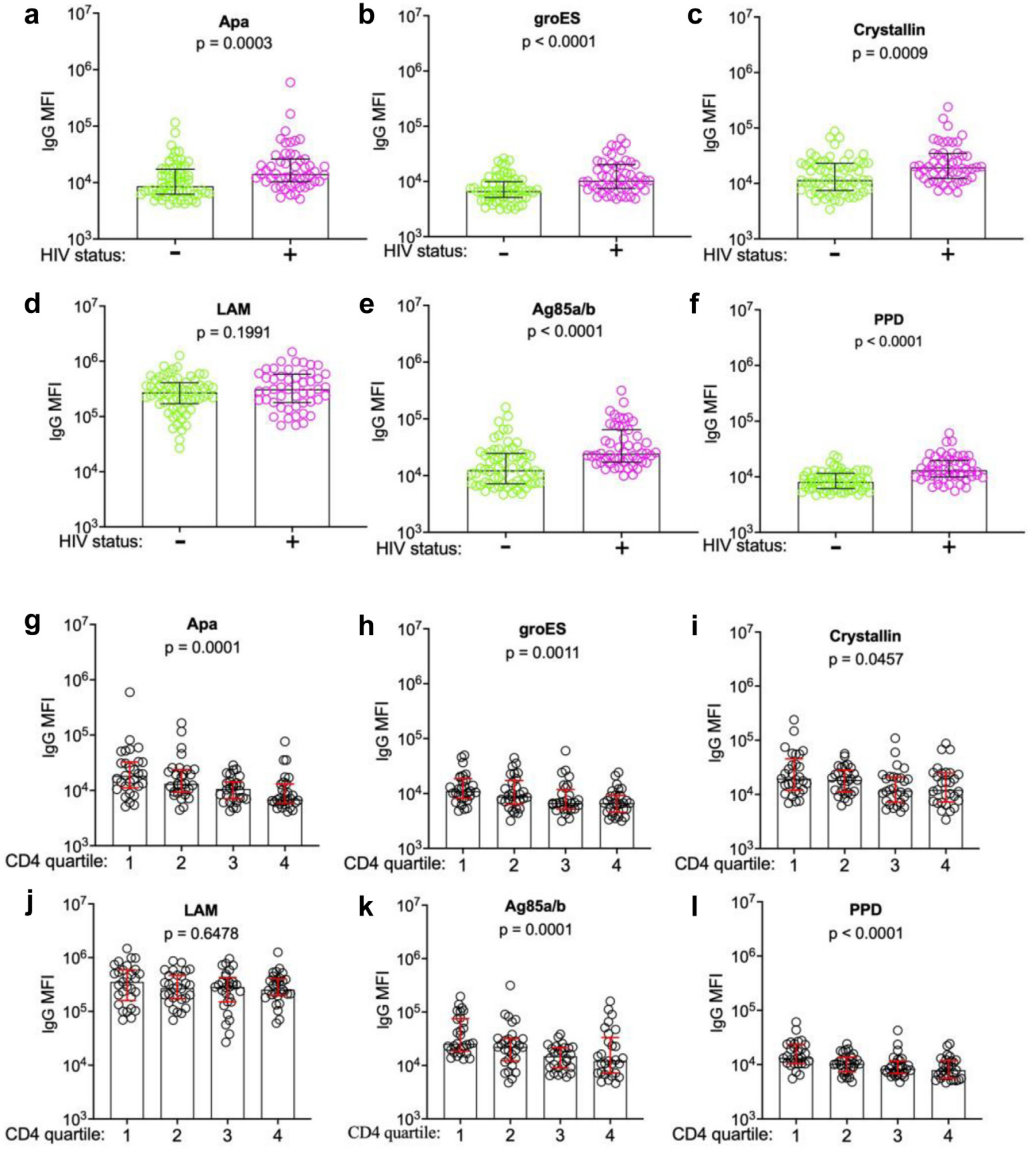
**Higher *Mtb*-specific total IgG in people with HIV and low CD4 T cell counts**. Relative concentrations (MFI) of total IgG specific to **a.** alanine- and proline-rich secreted protein (Apa), **b.** groES, **c.** alpha-crystallin, **d.** lipoarabinomannan (LAM), **e.** Antigen 85 complex (Ag85), **f.** Purified protein derivative (PPD) stratified by HIV status (people without HIV (n = 64, green), people with HIV (n = 51, pink), Mann–Whitney *U* test of difference between groups) and **g–l.** by CD4 count quartiles (Kruskal–Wallis test for difference across quartiles).

To understand whether the observed patterns for ESAT-6/CFP-10-specific IgG relative concentrations were consistent for other anti-*Mtb* IgG antibodies, we next measured antibodies to 6 other well-characterized and immunogenic *Mtb* antigens (Apa, GroES, alpha-crystallin, Ag85a/b, PPD, LAM. Antibody responses were analysed by HIV-status and CD4 cell count quartile. Five of the 6 measured *Mtb*-specific IgG responses were significantly increased in people with HIV, including Apa-specific IgG (*p* = 0.0003), GroES-specific IgG (*p* < 0.0001), Crystallin-specific IgG (*p* = 0.0009), Ag85a/b-specific IgG (*p* < 0.0001), anti-PPD IgG (*p* < 0.0001). LAM-specific IgG was the only *Mtb*-specific IgG that did not differ by HIV status (*p* = 0.1991) (Fig. 3a–f). Similarly, 5 of the 6 measured *Mtb*-specific antigens significantly differed across CD4 count quartile (Apa IgG-specific (*p* = 0.0001), GroES-specific IgG (*p* = 0.0011), Crystallin-specific IgG (*p* = 0.0457), Ag85a/b-specific IgG (*p* = 0.0001), PPD-specific IgG (*p* < 0.0001)), with a LAM-specific IgG again the exception (*p* = 0.7146, Fig. 3g–i).

### Non-specific hypergammaglobulinemia contributes to, but does not entirely account for the high relative concentration of anti-*Mtb* antibodies in people with HIV and low CD4 cell counts

**Fig. 4:**
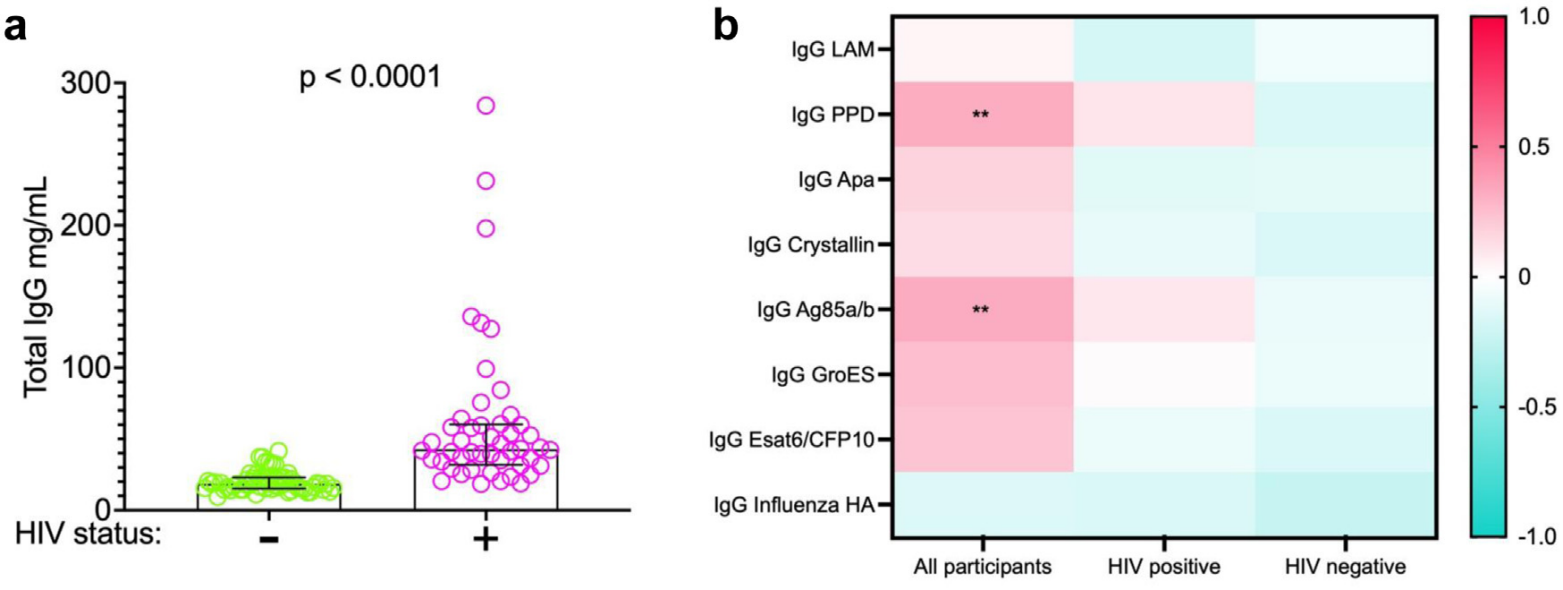
**Relationship between total IgG and *Mtb*-specific IgG**. **a.** Total IgG concentration (mg/mL), by HIV status (n = 115, people without HIV (n = 64, green), people with HIV (n = 51, pink), Mann–Whitney *U* test of difference between groups). **b.** Heatmap showing correlations between *Mtb*-specific IgG relative concentrations (MFI) and total IgG concentrations (mg/mL) (blue indicates negative correlation and pink indicates positive correlation) and level of significance (∗∗ for *p* < 0.001, Benjamini-Hochberg correction for multiple comparisons).

Hypergammaglobulinemia is a known consequence of HIV infection and is driven by non-specific activation of T cells and plasma cells.^19^^,^^20^ To determine whether this accounted for the observed results, we used a quantitative ELISA to measure total IgG in all study participants. Total IgG concentrations were significantly higher in participants with HIV compared to the participants without HIV (*p* = 0.0001) (Fig. 4a), consistent with hypergammaglobulinemia in the group with HIV. We next correlated total IgG concentration with *Mtb*-specific IgG antibody relative concentrations in all participants, and in participants grouped according to HIV status (Fig. 4b). When evaluated across all participants, there was a trend towards positive correlation correlations between total IgG and multiple specific anti*-Mtb* antigens IgGs but only 2 of the *Mtb* antigens (i.e. PPD and Ag85ab) showing significant positive correlation (Fig. 4b). The absence of correlation of total IgG with influenza HA-specific IgG, in contrast to positive correlations of multiple *Mtb* antigens, suggests an *Mtb*-specific process that is not adequately explained by hypergammaglobulinemia alone. However, when participants were stratified by HIV status, there was no significant correlation between total IgG and any *Mtb*-specific antibodies, suggesting that those correlations observed in the unstratified group were driven by HIV status. Thus, HIV-mediated hypergammaglobulinemia does not appear to entirely explain the increased relative concentration of *Mtb*-specific IgG found in individuals with HIV in this cohort.

### Lowest ESAT-6/CFP-10 IFN-γ production but highest ESAT-6/CFP-10 IgG in people with HIV and low CD4 cell counts

**Fig. 5:**
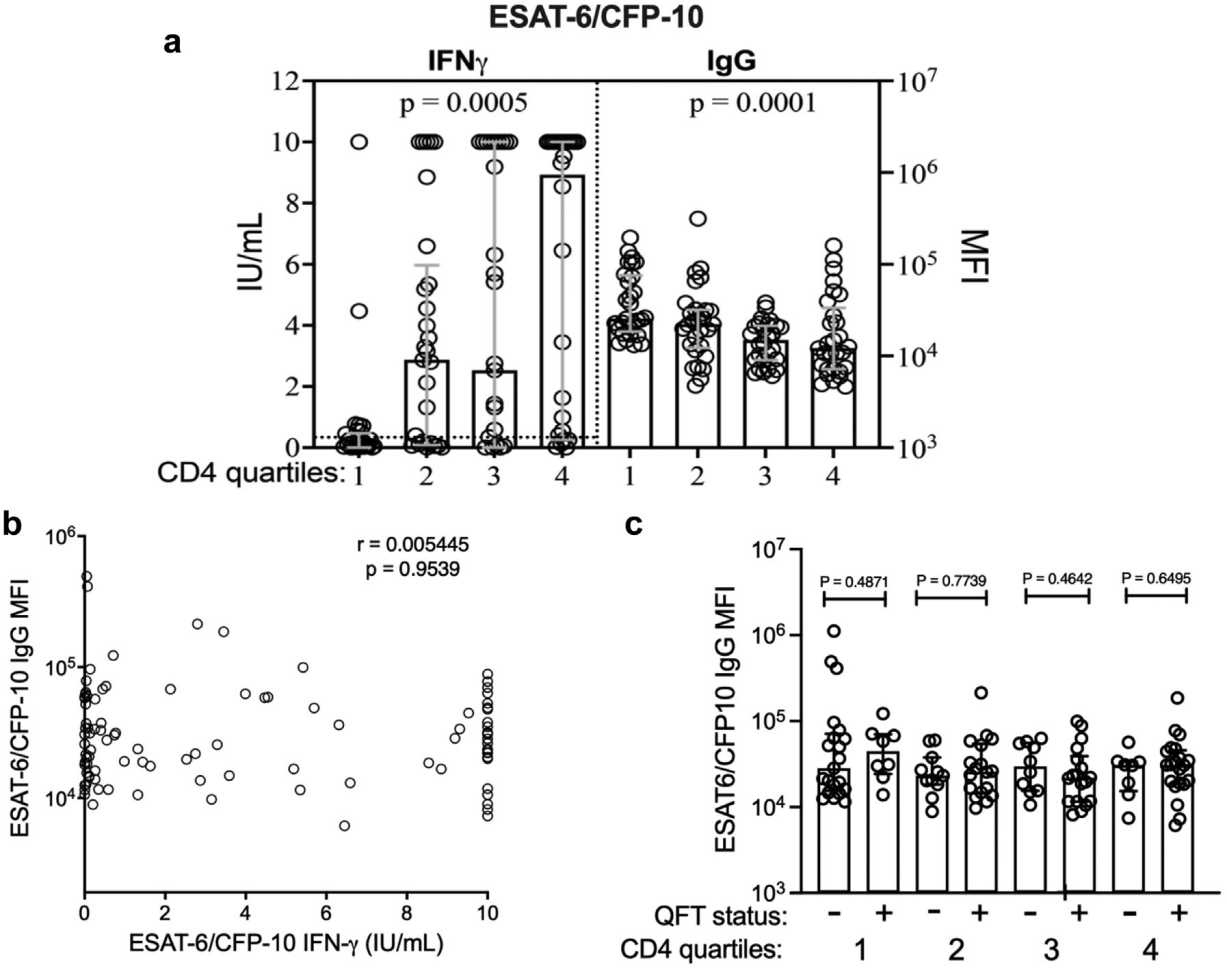
**Discordant ESAT-6/CFP-10-specific IFN-γ and IgG**. **a.** Left panel: ESAT-6/CFP-10 specific IFN-γ concentration (IU/mL) stratified by CD4 count quartiles (n = 118, Kruskal–Wallis test for difference between quartiles, dotted line represents the positive test cut off (≤0.35 IU/mL) for the QFT assay). Right panel: Relative concentration of ESAT-6/CFP-10-specific total IgG stratified by CD4 count quartiles (n = 115, Kruskal–Wallis test). **b.** ESAT-6/CFP-10 specific IFN-γ concentration (IU/uL) vs. ESAT-6/CFP-10-specific total IgG relative concentration (MFI, n = 115, Spearman's test of correlation) **c.** ESAT-6/CFP-10 IgG relative concentration (MFI) stratified by QFT status within CD4 count quartiles (n = 115, Mann–Whitney *U* test of difference between groups).

The QuantiFERON assay measures *Mtb*-specific T cell immune response to specific *Mtb* antigens ESAT-6/CFP-10 by quantifying IFN-γ produced in response to these antigens. Measurement of ESAT-6/CFP-10-specific IFN-γ and IgG in the same individuals, stratified by CD4 cell count quartile demonstrated that people in the lowest CD4 count quartile produced the least *Mtb*-specific IFN-γ (*p* = 0.0005) and the highest relative concentration of *Mtb*-specific IgG (*p* = 0.0001) (Fig. 5a). Direct comparison of these two measures, however, showed no correlation between ESAT-6/CFP-10 IFN-γ production and relative concentrations of ESAT-6/CFP-10 IgG among all participants (Fig. 5b) and no difference in relative ESAT-6/CFP-10 IgG concentrations between participants with positive and negative QTF results when stratified into CD4 count quartiles (Fig. 5c). Among participants with relatively preserved CD4 counts (3rd and 4th quartiles), comparison of the relative concentrations of the other *Mtb*-specific antigens IgG between participants with positive and negative QTF results also showed no differences (Supplementary Fig. 2).

## Discussion

In a group of well characterized people without HIV and antiretroviral-naïve people with HIV from KZN, South Africa we identified discordant results between *Mtb*-specific IFN-γ and *Mtb*-specific IgG, which was most striking in HIV-positive people with low CD4 cell counts. We found that 58% of adults in our study population had evidence of latent *Mtb*, as defined by a positive QFT test. Male sex, older age and higher CD4 count were associated with QFT positivity. In contrast, in the same group of people, ESAT-6/CFP-10*-*specific IgG was significantly higher in people who were HIV-positive and had low CD4 cell counts. Notably, in participants in the lowest CD4 count quartile, ESAT-6/CFP-10-specific IFN-γ release was lowest and the relative concentration of anti-ESAT-6/CFP-10*-*specific IgG antibodies was highest (Fig. 5a). Antibodies to 6 of 7 Mtb-specific antibodies followed this same pattern, while a control antibody specific to the hemagglutinin protein of influenza, did not. These results suggest that the QFT assay may underestimate LTBI among immunosuppressed people with HIV and *Mtb*-specific IgG may be a useful alternative biomarker for *Mtb* infection.

The current gold standard for the diagnosis of LTBI are interferon gamma release assays like the QFT, which rely on host T cell function to produce IFN-γ in response to *ex vivo* stimulation. In our study HIV status was not an independent modulator of QFT positivity, while CD4 count was. By stratifying participants into CD4 count quartiles, we observed that PLWH with relatively preserved CD4 counts (429–923 cells/mm^3^) had higher QFT positivity when compared to individuals without HIV in the similar CD4 counts range. Quantitative analysis of IFN-γ production in response to ESAT-6/CFP-10 showed that participants with HIV in the lowest CD4 quartile produced significantly lower IFN-γ compared to the participants with HIV in the higher CD4 count quartiles. In the absence of plausible explanation for why people with lower CD4 cell counts would have less exposure to *Mtb* infection, it is likely that these results are due to CD4 T cell depletion and/or exhaustion causing false-negative QFT tests in this group. This may also be the result of the QFT assay being performed on a fixed volume of whole blood; a limitation that is addressed in ELISpot assays, like the T-SPOT. TB test that use a set number of peripheral mononuclear cells per assay and therefore may be less affected by peripheral CD4 cell depletion.^21^ Although measures of *Mtb*-specific IFN- γ production have been shown to perform better than the tuberculin skin test in people living with HIV,^21^^,^^22^ our QFT results are in line with previous studies that have found lower rates of IGRA positivity in people with lower CD4 T cell count^12^^,^^23^ and PLWH.^24^^,^^25^

We also found that increasing age was an independent predictor of QFT positivity consistent with cumulative *Mtb* exposure over years of life.^23^^,^^26^ Male sex was also an independent predictor of QFT positivity in this study and is consistent with reports from other settings.^27^^,^^28^ The mechanisms for higher rates of *Mtb* infection and TB disease in males is not precisely defined, but is thought to be due to a combination of social factors that may promote *Mtb* exposure, such as environmental and occupational exposures and tobacco use, and increased biological susceptibility due to the differential effects of sex hormones on TB immunity.^27^, ^28^, ^29^, ^30^

IgG antibodies against *Mtb*-related antigens were considered potentially useful markers for *Mtb* infection over a decade ago, but further development of diagnostic modalities utilizing them was discouraged by WHO (2012) in favour of using the available methods at that time.^31^ Here, using a customized Luminex assay,^17^ we found that individuals with HIV had higher relative concentration of ESAT-6/CFP-10 IgG compared to people without HIV; this finding contrasted with the similar QFT results observed in groups with and without HIV. Older age and decreased CD4 count were also associated with increased ESAT-6/CFP-10-specific IgG, congruent with the QFT results. For QFT, we observed the lowest rates of positivity in individuals in the lowest CD4 count quartile, in contrast to the *Mtb*-specific plasma circulating anti-ESAT-6/CFP-10-specific IgG antibodies which had the highest concentration in this group (*p* = 0.0001) (Fig. 5). Antibodies to 5 of the 6 Mtb antigens assessed followed the same pattern as anti-ESAT-6/CFP-10-specific IgGs while the non-*Mtb*-related IgG control (the hemagglutinin protein of influenza) did not vary between HIV or CD4 groups, suggesting that the finding of higher *Mtb*-specific antibodies was pathogen-specific. Interestingly, LAM-specific IgG was not influenced by HIV infection or low CD4 count. A possible reason for this is that LAM is not specific to *Mtb*, and is found in the cell wall of other species of *Mycobacterium* that are ubiquitous in the environment.^32^ Although increased levels of PPD-specific memory B cells and IgG have been reported after BCG vaccination,^33^^,^^34^ it is unlikely that BCG vaccination explains these results because ESAT-6 and CFP-10 are proteins in the RD-1 region that is specific to *Mtb*. Interestingly, the concentration of ESAT-6/CFP-10 IFN-γ and relative concentration of ESAT-6/CFP-10 IgG did not correlate in all participants nor in those participants with preserved CD4 counts (Fig. 5b and c), indicating that circulating ESAT-6/CFP-10 IgG is not a direct biomarker for QuantiFERON status. Possible reasons for this may include technical limitations of the currently available assays and/or biological heterogeneity of the B and T cell responses to *Mtb* infection. However, recent data in household contacts of individuals with active TB suggest that *Mtb*-specific antibodies may capture *Mtb* exposure that is not reflected in their QFT testing.^17^ Indeed, TST and IGRA testing may not be adequate gold standards for the spectrum of *Mtb* exposure and infection conventionally thought of as LTBI.^3^ Comprehensive profiling of the *Mtb* antibody response, including additional antigens, isotypes, subclasses, Fc modifications, and compartments, will be key in elucidating whether humoral responses can offer resolution of *Mtb* exposure, infection, and disease beyond that measured by traditional diagnostic testing.

Hypergammaglobulinemia which has been linked to HIV infection^19^ may be a non-specific reason for elevated *Mtb*-specific antibodies in PLWH. Consistent with prior literature, in this study, total IgG titers were higher in HIV-positive participants. Hypergammaglobulinemia may partially explain higher anti-*Mtb* antibodies in PLWH, but we observed contrasting patterns between hemagglutinin-specific (*Mtb* non-specific) and *Mtb*-specific IgG, indicating that hypergammaglobulinemia alone does not explain higher anti-*Mtb* antibody relative concentrations in those with low CD4 counts. Further experiments with independent and larger cohorts are required to determine whether elevated anti-*Mtb*-related IgG can be detected independent of or when controlling for generalized hypergammaglobulinemia and whether detection of *Mtb*-specific antibodies may be a useful diagnostic strategy, particularly in HIV-positive participants with low CD4 counts. Overall, our results point to a potential role for *Mtb*-specific antibodies to provide a way of defining *Mtb*-infection status that is not dependent on T cell function.

This study has several limitations. These data were collected from a convenience sample and are therefore not population representative, which limits generalizability. All individuals with HIV assessed were antiretroviral therapy naïve, which also limits generalisability. There was a small but significant age difference between the people living with HIV and the people without HIV, which may have confounded observed differences in measures of *Mtb*-infection. Limitations of the data include the lack of positive/negative cut-off thresholds or absolute quantification for the bead-based *Mtb*-specific IgG assays used here. The assay also measures relative concentration rather than an absolute quantitative measure of concentration that would allow direct comparison to total IgG concentrations, which would be required to address the contribution of hypergammaglobulinemia more definitively. Future work that combines well-defined PLWH (including people on antiretroviral therapy) and individuals without HIV from areas of high and low TB endemicity will be needed to advance the evaluation of *Mtb*-specific IgGs, especially antibodies to RD-1 proteins (to avoid cross reactivity with BCG vaccination) as potential biomarkers for LTBI detection. Once the most promising antibody candidates for biomarker use are identified, it will be important to develop quantitative tests for measurement of their absolute concentration (e.g. ELISA assays) to advance evaluation of their diagnostic and prognostic performance. While BCG vaccination history of study subjects was not recorded, most individuals in South Africa receive BCG vaccination in childhood^35^ raising the concern that we may be detecting BCG-specific, rather than *Mtb*-specific, antibodies and is a potential limitation to this study. Although studies of BCG vaccination in humans and macaques do demonstrate increased *Mtb*-specific antibodies across isotypes,^34^^,^^36^^,^^37^ these titers appear to wane to background levels within months of vaccination and are unlikely to persist into adulthood. Moreover, ESAT-6 and CFP-10 are not present within BCG, and thus our observation of antibody responses to these antigens likely reflects true *Mtb* exposure.

Even with its limitations and possible under-estimate of LTBI in people with HIV and low CD4 counts, our finding of QFT-confirmed LTBI in 58% of 18–50 years old adults is on the higher side of what has been reported in South African populations. In a previous study using TST, only 34.3% of adults in South African urban townships around Johannesburg were reported to have LTBI.^26^ Mathematical models estimation using TST data supports the above results, reporting 30%–40% South Africans having LTBI according to those estimations.^38^ A TST survey among gold miners in Johannesburg showed LTBI rates as high as 85%, which correlates with the higher rates of active TB cases identified within gold miners.^39^ A survey in a Cape Town township showed that about 40%–50% of adolescents had LTBI using QFT assay,^40^ in line with the rates we report here. However, a survey of adolescents in rural KZN using QFT showed a much lower rate of 23% LTBI.^41^ With high rates of latent TB infection, South Africa is prioritizing preventative therapies, and to this end less expensive, easier and more accurate tests for LTBI especially in PLWH are urgently needed.^42^

Overall, our data suggest high rates of latent TB infection among adults with and without HIV and that the QFT assay likely underestimates LTBI in people with HIV and low CD4 T cell counts. Eliminating TB remains challenging due to the limitations in diagnosing TB at various stages along the infection spectrum.^2^ Furthermore, while increasing access to TB preventative therapy is a global priority, very limited screening for LTBI in South Africa has been done, in part because of the indirect nature and complexity of interpretation required by the current assays. *Mtb*-specific IgG antibodies may hold promise as alternative biomarkers for LTBI, especially in people with low CD4 count.

## Contributors

MM, TN and EBW designed the overall study. ZM, DR and FK collected the clinical samples. MM, TB and SK performed the QuantiFERON assays. KAB and LRLD performed the antibody assays. MM, EBW, KB, LRLD, GA and TN verified data and performed the data analyses and interpretation, and LM validated all the statistical methods used. EBW, TN and GA provided supervision. MM, TN, and EBW wrote the manuscript. All authors reviewed the manuscript and approved the final version.

## Data sharing statement

Deidentified participants raw data used to generate results for this work is available on the following online figshare repository: https://doi.org/10.6084/m9.figshare.21701942.v3.

## Declaration of interests

All authors declare that they have no conflict of interest.
