# Supplementary material for: Discrepancy between Mtb-specific IFN-γ and IgG responses in HIV-positive people with low CD4 counts
Source: eBioMedicine. 2023 Mar 2;90:104504. doi: 10.1016/j.ebiom.2023.104504 (PMC9996381; doi:10.1016/j.ebiom.2023.104504)
Supplement: Supplementary Table S1 [file mmc1.pdf]

Supplementary Table 1: Detailed information on all reagents used in the study.

Product name	Product type	Supplier name	Catalogue number
QFT-TB Gold Plus Bulk Tubes (50)	Blood collection tubes	Qiagen: Baker medical (South African local supplier)	622536
QFT-Plus 2 Plate Kit ELISA (44)	QFT assay kit	Qiagen: Baker medical (South African local supplier)	622120
QuantiFERON Control Panel	Cytokine	Qiagen: Baker medical (South African local supplier)	0594-0805
PPD	Antigen	Staten Serum institute (SSI)	-
Ag85ab	Antigen	BEI Resources	NR-14855
ESAT-6	Antigen	BEI Resources	NR-49424
CFP-10	Antigen	BEI Resources	NR-49425
Apa	Antigen	BEI Resources	NR-14862
GroES	Antigen	BEI Resources	NR-14861
Crystallin	Antigen	BEI Resources	NR-14861
LAM	Antigen	BEI Resources	NR-14848
Flu/HA	Antigen	ImmuneTech	-
