# Supplementary material for: Discrepancy between Mtb-specific IFN-γ and IgG responses in HIV-positive people with low CD4 counts
Source: eBioMedicine. 2023 Mar 2;90:104504. doi: 10.1016/j.ebiom.2023.104504 (PMC9996381; doi:10.1016/j.ebiom.2023.104504)
Supplement: Supplementary Figures S1 and S2 [file mmc2.pdf]

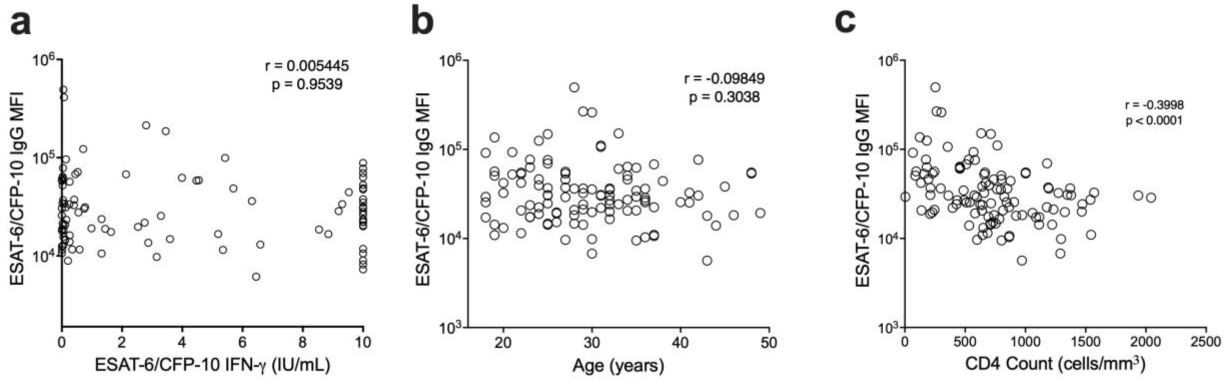

Supplementary Figure 1: Correlation of ESAT-6/CFP-10-specific IFN-γ and IgG with CD4 count and age. a. ESAT-6/CFP-10 specific IFN-γ concentration (IU/uL) vs. CD4 cell count (cells/mm^3, n=118, Spearman's test of correlation). **b.** ESAT-6/CFP-10-specific total IgG relative concentration (MFI) vs. age (years, n=115, Spearman's test). c.ESAT-6/CFP-10-specific total IgG relative concentration (MFI) vs. CD4 cell count (cells/mm^3, n=115, Spearman's test).

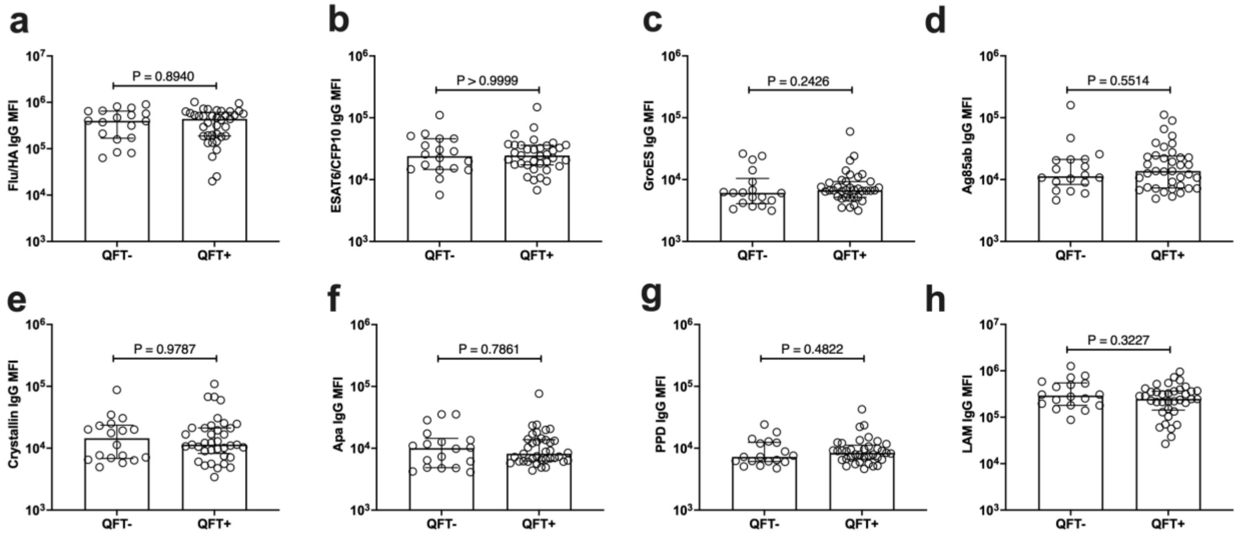

Supplementary Figure 2: *Mtb*-specific IgG relative concentrations by QuantFERON status. Relative concentration of **a.** Flu/HA, **b.** ESAT-6/CFP-10, **c.** GroES, **d.** Ag85a/b, **e.** Crystallin, **f.** Apa, **g.** PPD, **h.** LAM-specific total IgG (MFI) among participants with relatively high CD4 cell counts (3rd and 4th quartile, n=55), stratified by QuantiFERON (QFT) status (Mann-Whitney U test of differences between groups).
